# Privacy, ethics, transparency, and accountability in AI systems for wearable devices

**DOI:** 10.3389/fdgth.2025.1431246

**Published:** 2025-06-17

**Authors:** Petar Radanliev

**Affiliations:** ^1^ Department of Computer Science, University of Oxford, Oxford, United Kingdom; ^2^ Alan Turing Institute, London, United Kingdom

**Keywords:** wearable technology, artificial intelligence, machine learning, data privacy, ethical considerations, health data science, digital identity systems

## Abstract

The integration of artificial intelligence (AI) and machine learning (ML) into wearable sensor technologies has substantially advanced health data science, enabling continuous monitoring, personalised interventions, and predictive analytics. However, the fast advancement of these technologies has raised critical ethical and regulatory concerns, particularly around data privacy, algorithmic bias, informed consent, and the opacity of automated decision-making. This study undertakes a systematic examination of these challenges, highlighting the risks posed by unregulated data aggregation, biased model training, and inadequate transparency in AI-powered health applications. Through an analysis of current privacy frameworks and empirical assessment of publicly available datasets, the study identifies significant disparities in model performance across demographic groups and exposes vulnerabilities in both technical design and ethical governance. To address these issues, this article introduces a data-driven methodological framework that embeds transparency, accountability, and regulatory alignment across all stages of AI development. The framework operationalises ethical principles through concrete mechanisms, including explainable AI, bias mitigation techniques, and consent-aware data processing pipelines, while aligning with legal standards such as the GDPR, the UK Data Protection Act, and the EU AI Act. By incorporating transparency as a structural and procedural requirement, the framework presented in this article offers a replicable model for the responsible development of AI systems in wearable healthcare. In doing so, the study advocates for a regulatory paradigm that balances technological innovation with the protection of individual rights, fostering fair, secure, and trustworthy AI-driven health monitoring.

## 1 Introduction

The proliferation of devices capable of continuous physiological monitoring, ranging from consumer-grade smartwatches to clinically validated biosensors, has enabled the capture of vast volumes of personal health data at an unprecedented scale and temporal resolution. When analysed through advanced algorithmic techniques, these data streams offer considerable promise in advancing diagnostic accuracy, facilitating personalised interventions, and supporting population-level health insights.

Yet the very features that render these systems powerful also give rise to a complex set of ethical, legal, and epistemological challenges. The passive and pervasive nature of data collection, the opacity of model inference, and the risk of algorithmic discrimination all call into question the adequacy of existing regulatory frameworks. In particular, the conflation of technical sophistication with clinical utility has, in some cases, obscured the normative dimensions of automated health decision-making. This is especially pertinent where decisions are derived from models that are neither explainable to end-users nor fully auditable by developers, thereby eroding the conditions necessary for trust, autonomy, and accountability. This raises many ethical issues on data privacy, consent, bias and fairness, security vulnerabilities (1), and poses significant risks if misused or inadequately protected (2).

A growing body of scholarship has articulated the need for ethical and regulatory reform in this domain. However, much of the discourse remains either abstractly principled or overly reactive to high-profile failures. What remains underdeveloped is a systematic, implementable framework that aligns the affordances of data-driven methodologies with the imperatives of transparency, fairness, and regulatory compliance, particularly in the context of wearable systems operating in health-sensitive environments.

This article seeks to address that gap. It offers a critical examination of the ethical tensions inherent in AI-enabled wearables and introduces a new methodological framework that operationalises core principles of responsible AI. By embedding transparency and accountability throughout the data lifecycle, from collection and preprocessing to modelling and deployment, the framework provides a normative and technical structure for aligning innovation with public values. In doing so, this study contributes to the field of health informatics but also to the broader discourse on the governance of emerging technologies in data-rich societies.

## 2 Wearable sensors and the importance of ethics and privacy in the AI and ML algorithms

Wearable sensors and smart devices have become increasingly popular and are integral to personal data collection (3). These devices monitor our health metrics (4), behavioural patterns (5), and location in real-time (6). As these devices become more interconnected with AI and ML systems (7), the implications for privacy and ethics increase (8). Wearable sensors collect a vast amount of personal data, which can be helpful for personal health and productivity (9). However, when fed into AI and ML algorithms, this data can be used to profile individuals without explicit consent. This can lead to invasive targeted advertising, increased insurance premiums based on health data, or even surveillance by governments or companies.

AI and ML models often rely on large datasets to improve their accuracy (10). An ethical concern arises when users don't give explicit, informed consent for their data to be used in such ways (11). AI and ML algorithms, when trained on data from wearable sensors, could inadvertently perpetuate or even exaggerate societal biases (12). For instance, if a specific demographic primarily uses wearables, algorithms could be biased towards that demographic, leading to discrimination against underrepresented groups (7, 11, 13, 14).

Integrating wearable sensors and AI systems presents an attractive target for cyber-attacks (15). Personal data, especially health data, is valuable (16). A breach violates individual privacy and can lead to identity theft or financial loss (17). As wearable sensors and AI systems advance, there is also a risk of reducing human autonomy as individuals may become too reliant on these systems (3). This over-reliance on algorithms could lead to a scenario where people are consistently guided by what the AI thinks is best for them, potentially eroding human agency and decision-making.

**Figure 1:**
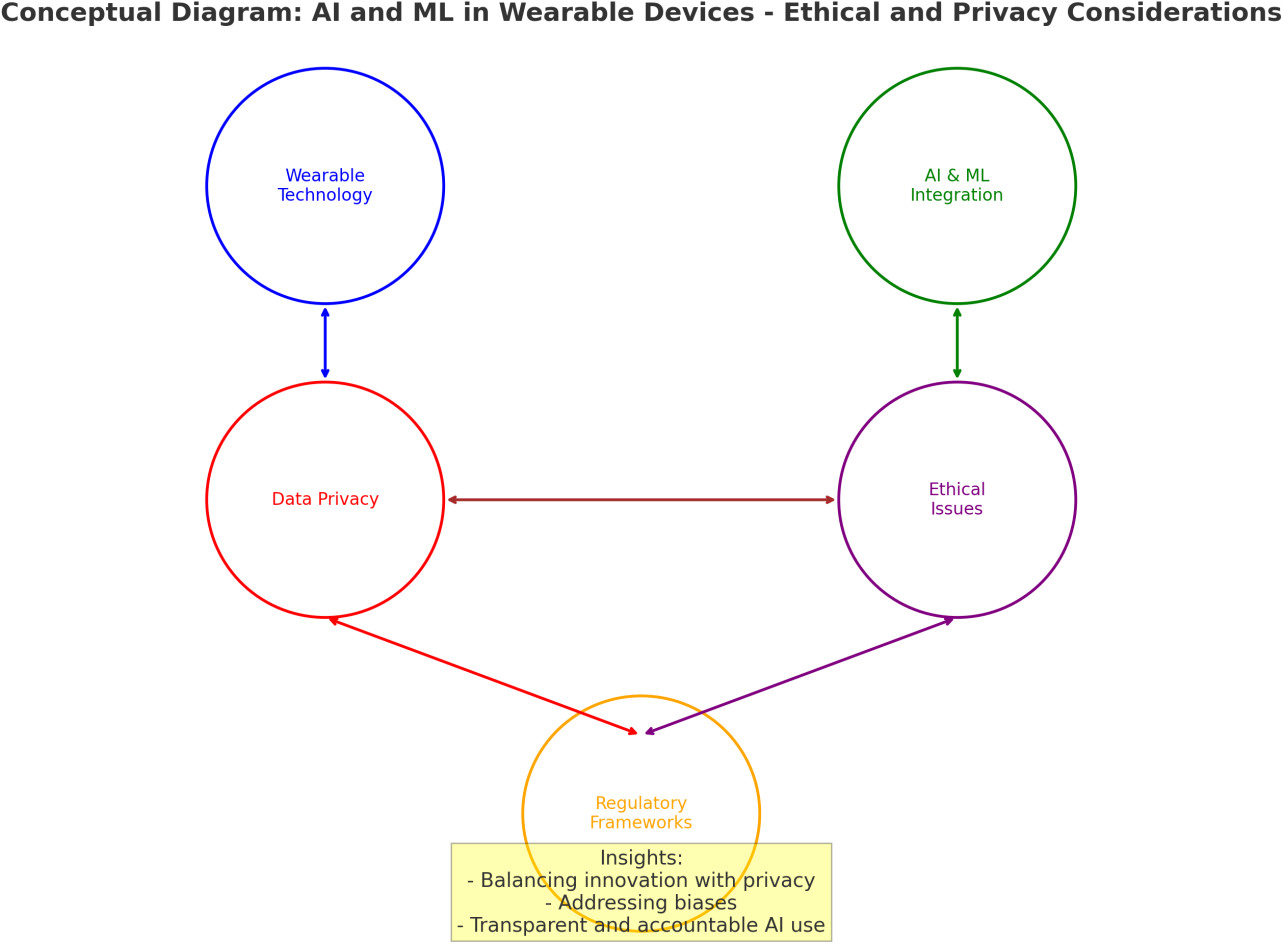
The key risks involved in data collection and surveillance, consent and awareness, bias and discrimination, data security and breaches.

Figure 1 describes how wearable sensors are integral to the broader conversation on ethics and privacy in AI and ML. The potential risks involved in data collection and surveillance, consent and awareness, bias and discrimination, data security and breaches, and depersonalisation and autonomy must be carefully considered to protect personal privacy and independence while securing the benefits of these technologies. Figure 1 presents a conceptual framework outlining key ethical and privacy risks associated with AI and ML integration in wearable sensor technologies. While the figure captures core challenges, such as data collection risks, consent ambiguity, algorithmic bias, and data breaches, it is important to foreground the foundational role of *transparency* within this ecosystem. Transparency can be seen as an auxiliary principle, but also as an essential precondition for enabling accountability, user trust, and regulatory compliance in AI systems. As articulated in current literature on AI ethics discussed earlier in this section, transparency allows stakeholders to understand how decisions are made by AI models and is integral to mitigating harms stemming from opaque algorithmic processes. Moreover, transparency operates at the system design level ensuring traceability and explainability, and at the user interface level, where individuals must be able to interpret and challenge automated decisions. These considerations provide the rationale for incorporating transparency explicitly in any discussion of ethical AI in wearables.

In Figure 1 it can be seen that transparency and control are essential for AI models operating in the background (18), as they can make decisions that profoundly affect individuals. Users have a right to understand how these decisions are made and to have control over their data (19). Therefore, companies developing wearables interlinked with AI should prioritise transparent operation and give users more granular control over their data (20). While wearable sensors offer personalised healthcare, convenience, and enhanced productivity (21), they also bring ethical concerns, particularly when integrated with AI and ML systems. The constant collection of data by these devices requires transparency, and accountability needs to be regulated in terms of ethics and privacy (22).

*Transparency* underpins the framework in Figure 1, and informs the progression of ethical risk toward the final mitigation. Transparency acts as a mediating construct that intersects with each identified risk, whether through clarifying data flows, exposing algorithmic logic, or enabling consent to be informed and revocable. Its omission as a discrete visual element may inadvertently obscure its structural significance, particularly in relation to established regulatory standards such as the GDPR and the EU's Ethics Guidelines for Trustworthy AI, both of which cite transparency as a central principle. Accordingly, the model should be interpreted with the understanding that transparency is embedded throughout the flow, guiding the operationalisation of ethical governance at each stage. By expressing transparency as an embedded principle and a regulatory imperative, the framework aligns with international standards and supports business transformation strategies that prioritise explainability, fairness, and user empowerment.

## 3 Wearable sensors and health data science

**Figure 2:**
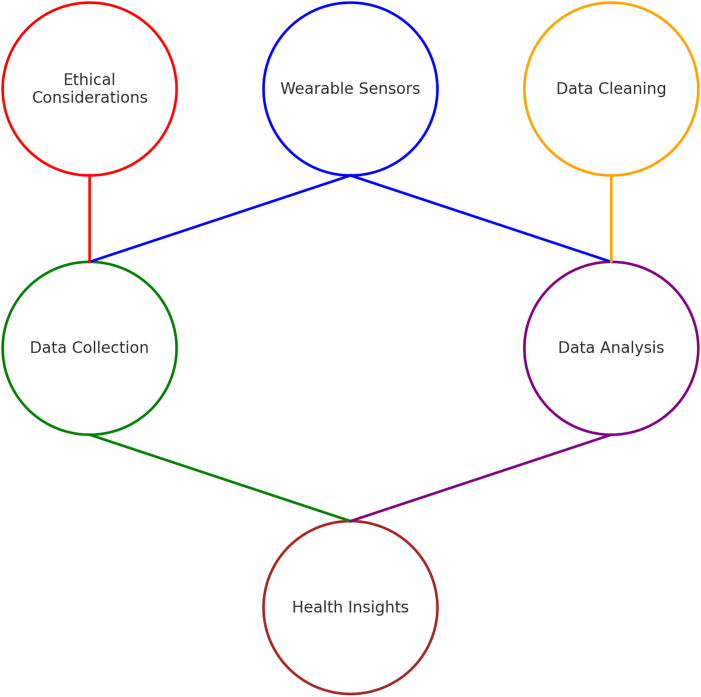
The interconnectedness of various wearable sensors and health data science components.

Wearable sensors like fitness trackers, smartwatches, and medical devices are essential for collecting physiological signals to assess health (3). These signals include heart rate, sleep patterns, and more. Accelerometers in wearable devices measure acceleration forces that help monitor physical activity, sleep patterns, and more (21). In Figure 2, the process of integrating wearable sensors into health data sciences is defined. This relationship is then discussed in relation to validating datasets for privacy, ethics, transparency, and accountability in AI systems for wearable devices.

Figure 2 illustrates the technical process by which data from wearable sensors is transformed into structured datasets for AI and ML model development. This pipeline (comprising stages of collection, cleaning, and analysis) is foundational for developing predictive models in health data science. However, transparency is a critical yet implicit element in this workflow, one that requires explicit acknowledgment. The literature emphasises that data provenance, preprocessing steps, and model interpretability must be made transparent to ensure reproducibility, trust, and compliance with regulatory requirements (discussed in the review of literature). While the US has taken more business and innovation friendly approach (23) to AI ethics (24, 25), from the EU perspective, transparency should be viewed not as a best practice but as a regulatory obligation, mandated by frameworks such as the EU/UK General Data Protection Regulation (GDPR) (26, 27) and the AI Act (28, 29), which require that data subjects be informed of how their data is processed and for what purpose. In this context, transparency bridges the technical data pipeline and the ethical imperatives that govern its application.

Figure 2 outlines the stages of data preparation with a visual or textual representation of *transparency*, and its central role in mediating each stage of the process. Without transparency, the progression from raw sensor data to final analysis lacks the traceability required by modern data governance standards. For instance, under Article 5 of the GDPR, the principles of lawfulness, fairness, and transparency require that data handling processes be documented and communicated in an intelligible form to end users. Similarly, the EU's Ethics Guidelines for Trustworthy AI identify transparency as one of seven key requirements, insisting on traceable data flows and explainable AI outputs. Therefore, Figure 2 should be interpreted with the understanding that transparency is an embedded, cross-cutting requirement that substantiates each phase of data handling and model training. Its inclusion is not merely ethical, but structurally necessary for regulatory compliance and public accountability in health-focused AI systems. Figure 2 also defines the process of validating datasets. One example of such a validation dataset is the MobiFall (30) and MobiAct (31) datasets, which contains accelerometery data from different real-world scenarios. Before applying AI and ML algorithms, the data collected from wearable sensors goes through three stages: collecting, cleaning, and analysing. In the collection stage, raw data is captured from the wearable devices. In the cleaning stage, noise is removed, and missing values are filled. In the analysis stage, data is structured for model training.

Deep Learning models (32), especially Convolutional Neural Networks (CNNs) (33), have efficiently processed sensor time-series data. Apple's smartwatch, for instance, uses complex algorithms, probably deep learning models, for its heart rate monitoring. Decision Trees and Random Forests are used for pattern recognition in wearable sensor data. These algorithms can be trained to detect abnormal patterns or behaviours. Support Vector Machines (SVM) can classify data into different categories, making them ideal for recognising different stages of activities or health conditions (34). One specific dataset used in this study as secondary data for this specific training and testing, is the DeepDance dataset (35). Even when using secondary data, ethical implications should be considered when using AI and ML algorithms with wearable sensor data. First, it's crucial to ensure that AI models are trained on diverse datasets to prevent biases. Wearable devices might yield skewed results if used in diverse populations but trained in a homogenous group. For example, a heart rate monitor might not be as accurate for individuals from specific ethnic backgrounds if the validation dataset lacks diversity.

Second, end-users need to understand how their data is being processed after the study is completed and their data is donated to an open-source repository. Transparency ensures trust, and accountability ensures there are mechanisms for redress if things go wrong. Wearable sensors collect intimate health data, making data encryption, anonymising datasets, and transparent data usage policies paramount. Fitbit has faced scrutiny regarding user data privacy concerns, which is an essential lesson for other wearable tech producers to prioritise data protection. This opens a new question on why we need ethics in AI systems (36).

## 4 Why ethics in AI?

Wearable sensors have experienced remarkable growth in recent years due to the rise in consumer health awareness. These devices collect vital health-related data, which can be used for various purposes, such as monitoring health, tracking activities, and predicting potential health issues. The first challenge with wearable sensors is correctly collecting data. The data collected could contain noise for various reasons, such as sensor misplacement, faulty readings, etc. Data cleaning becomes crucial to ensure the accuracy of any subsequent analysis.

Once the data is collected and cleaned, analysis is performed to derive meaningful insights. Depending on the complexity of the data, this involves using statistical methods, machine learning algorithms, or even deep learning techniques. For wearable sensor data, some popularly applied models include time-series analysis, convolutional neural networks (CNN) (37), recurrent neural networks (RNN) (38), random forests (39), and gradient-boosted trees.

Accelerometers are standard in many wearable devices. They help measure acceleration forces, which can be used to determine speed, direction, and orientation. Validation datasets like MobiAct (31) and UniMiB SHAR (40) commonly validate these accelerometers. These datasets provide standardised data that can be used to validate and calibrate accelerometer readings (41). Devices like Fitbit and Apple Watch use AI models to track heart rates, sleeping patterns, and other vital metrics, offering insights to the users. Some advanced wearables can predict potential health issues like cardiac arrests by analysing patterns in data. The use of AI in wearable sensors has made it possible to collect valuable data, make predictions, and provide personalised insights to users, thereby revolutionising the health and wellness industry.

Ethics in AI is essential as it significantly impacts our society (42). Ethical considerations ensure that AI technologies are developed and deployed responsibly, ensuring fairness, accountability, and transparency (11). AI can have profound societal implications, from job displacements due to automation to biases in decision-making systems. Without ethical considerations, AI can unintentionally reinforce societal inequalities and prejudices.

There are real-world examples where the lack of ethics in AI has caused harm (5), such as healthcare algorithms showing racial bias, facial recognition misidentifications, and privacy concerns with wearables (13). It is crucial to address ethical concerns head-on to harness the technology's full potential without compromising individual rights or societal values (6), especially as AI integrates more into healthcare and wearables.

## 5 Bias and fairness

Wearable sensors are electronic devices humans wear to monitor physiological and movement data. Some examples of wearable sensors are smartwatches like the Apple Watch and Fitbit, specialised wearables such as glucose monitors, and bright clothing embedded with sensors. In health data science, wearable sensors provide real-time and continuous data streams that can be used for various purposes, such as disease prediction and prevention, fitness and rehabilitation monitoring, sleep pattern analyses, and stress and mental health assessments.

Accelerometers are an essential component of many wearables, measuring movement data. However, accelerometer data needs to be validated to ensure reliability and interpretability. Researchers often use datasets like MobiAct and UniMiB SHAR to validate accelerometer data, which consist of annotated activities performed by participants. This allows researchers to correlate accelerometer readings with known actions.

Collecting, cleaning, and analysing wearable sensor validation datasets involves several steps. Firstly, data is collected from participants wearing the sensors during predefined activities. Then, smoothing, normalisation, and filtering are applied to ensure data quality. Finally, machine learning algorithms such as decision trees, random forests, and deep learning models are used in these datasets to predict and categorise user activities.

Bias in AI refers to systematic and non-random errors in predictions or decisions made by ML models. Biases can arise from various sources and inadvertently result in unfair or discriminatory outcomes (43). There are two types of bias: data bias and algorithmic bias. Data bias occurs when the training data does not adequately represent the population, resulting in skewed predictions. For instance, a wearable training dataset that primarily includes data from younger adults might perform less accurately for older populations. Algorithmic bias occurs when an algorithm's design or structure can introduce bias. A health tracking algorithm optimised for a specific demographic might perform differently for another.

Real-world examples of bias in wearables include heart rate monitoring bias and gender differences in caloric estimation. Wearables may be biased in their predictions due to differences in skin tone or basal metabolic rate, which can lead to discriminatory outcomes.

## 6 Consequences of bias

Wearable sensors are devices worn on the wrist, such as watches or patches, that continuously collect health metrics such as heart rate, body temperature, and movement patterns. They have been instrumental in remote patient monitoring, physical fitness assessment, and even diagnosing and predicting ailments.

Accelerometers are critical components in wearables that measure physical activity levels. Validating their readings is crucial to ensuring the reliability of the data collected. Therefore, it is imperative to have dependable datasets for validating accelerometers.

The NHANES datasets (41), developed by the National Health and Nutrition Examination Survey, is a comprehensive dataset commonly used for validating wearable sensor readings. This dataset contains accelerometer data and various health metrics, which help researchers compare and refine the accuracy of wearable sensor readings. Collecting, cleaning, and analysing wearable sensor data involves several steps. Collecting raw data from wearable sensors, especially from accelerometers, can be challenging due to the noise in the data. Strategies such as providing user guidelines for proper wear or redundant sensors are used to ensure consistent and reliable readings. Data cleaning is an important step that involves filtering out noise, addressing missing values, and rectifying inconsistent readings. Techniques like interpolation, outlier detection, or algorithms like Kalman filters are employed. The cleaned data is then analysed using machine learning models like Random Forest, Gradient Boosting Machines, or Neural Networks. These models help segment data, understand patterns, or predict health outcomes based on wearable sensor data, which are the consequences of bias in wearable AI (9).

Addressing biases in wearable AI systems to ensure accuracy, inclusivity, and fairness in health data science is important. Biases in datasets can cause wearables to be less accurate for certain groups, such as those outside of the age or ethnicity predominantly used in training datasets. For instance, facial recognition wearables have been known to misidentify individuals from certain ethnic backgrounds due to biases in training datasets. Biases can also lead to misrepresentation, such as an AI system trained predominantly on data from athletic individuals misrepresenting the health metrics of non-athletes.

To address these biases, diverse data collection is crucial to ensure that datasets include a variety of individuals in terms of age, gender, ethnicity, and health status. Additionally, machine learning techniques like adversarial training or re-sampling methods can help detect and counteract biases in training data. Manufacturers and researchers should also be transparent about potential limitations or biases in wearable devices to ensure transparent reporting.

## 7 Case study on bias

Wearable sensors are widely accepted as tools for continuous monitoring, providing valuable insights into an individual's health. These devices come in various forms, such as watches, bracelets, or patches, and can track several physiological parameters, such as heart rate, temperature, and motion.

One core component of many wearable devices is the accelerometer. It measures movement patterns, which can be interpreted for various health-related metrics. These metrics can validate datasets and provide additional insights into an individual's health. Analysing these datasets with AI and ML's help can lead to transformative possibilities in health data science.

Specific Datasets:

• MobiAct (31): A dataset that contains various activities performed by different age groups, making it diverse and suitable for a wide range of wearable application tests.
• UCI Human Activity Recognition Using Smartphones Dataset (44, 45): Features recordings of 30 subjects performing activities of daily living (ADL) while carrying a waist-mounted smartphone with embedded inertial sensors.
• WISDM (Wireless Sensor Data Mining) (46): Contains data from smartphone sensors, detailing activities like walking, jogging, standing, sitting, and more.
• HHAR (Heterogeneity Human Activity Recognition) (47, 48): This dataset includes smartphone and smartwatches data, with nine different activities being recognised.
• PAMAP2 Physical Activity Monitoring (49, 50): A dataset containing data from wearable sensors placed on different body parts. It records activities like walking, running, cycling, etc.
• Daphnet Freezing of Gait (51): Specifically designed for patients with Parkinson's Disease, this dataset collects data to recognise the “freezing” event in their gait.
• Actitracker (52–54): Collected from smartphone sensors, this dataset recognises six primary actions: jogging, walking, ascending stairs, descending stairs, sitting, and standing.
• Daily and Sports Activities (55, 56): This dataset captures 19 daily and sports activities using nine embedded sensors in a wireless body area network.
• Smartphone Dataset for Human Activity Recognition (HAR) in Ambient Assisted Living (AAL) (57): Focuses on indoor and outdoor activities using smartphone sensors.
• Opportunity Activity Recognition (58, 59): This dataset recognises human activities and their contextual aspects captured from various sensors placed around the body.
• CASAS (60): A multi-modal dataset for human activity recognition using smart home technologies.
• MSR Daily Activity 3D (61): This dataset uses depth maps captured from a depth camera, focusing on the daily activities of individuals.
• REALDISP Activity Recognition Dataset (62): It emphasises activity recognition using several sensors and realistic scenarios, emphasising the diversity of human actions.

Multidimensional process must be followed to prepare data from wearable sensors for AI models, which involves collecting, cleaning, and analysing the data (12). Each stage is critical in ensuring the quality and reliability of the model's predictions. Firstly, collecting data from wearable devices involves using APIs and SDKs or transmitting data wirelessly via Bluetooth, NFC, or Wi-Fi to a connected device or cloud storage. This process requires establishing a connection with the wearable device and requesting the desired data type before streaming or batch downloading it. Secondly, cleaning the collected data is essential because it can be messy due to device limitations, human errors, or external interference. This process involves filtering out noise and removing data points that don't meet certain quality criteria, such as outliers or missing data points. After cleaning the data, the next step is to analyse it using statistical methods or visualisations to identify patterns and trends. This analysis helps to uncover insights that can be used to train AI models for various wearable sensor applications and research angles.

Preparing wearable sensor data for AI models to achieve accurate and reliable predictions is important. A clear and concise flow of ideas is essential for effective communication. This involves organising the ideas logically, transitioning smoothly between sentences and paragraphs, and presenting the main ideas in a logical order. Transitional words and phrases can also help establish a smooth flow of ideas. To achieve this, it is important to plan and organise the content beforehand and revise and edit the text to ensure clarity and concision. If the data is transformed into image-like structures, we can use CNN models, for validation of the model on the test set, fine-tuning hyperparameters. Once we have verified the model's accuracy, we can deploy it for real-time analysis or batch processing of new data. By following these steps, researchers and developers can ensure the highest quality data interpretation, maximising the potential benefits of wearable sensors when combined with AI techniques.

### 7.1 Artificial intelligence and machine learning in wearable data processing

Machine learning models, particularly deep learning models such as CNNs and Recurrent Neural Networks (RNNs), are highly effective in processing time-series data typical in wearables. For instance, a study (63) used CNNs to categorise six different activities (walking, walking upstairs, walking downstairs, sitting, standing, and lying down) based on accelerometer and gyroscope data from smartphones, obtained from the UCI datasets (44, 49, 51, 55, 57, 58, 62).

However, biased data, particularly in health, can have serious consequences. For instance, suppose a hypothetical wearable was designed to monitor heart rates and predict heart diseases. If most initial training data was collected from a single ethnic group, the device might not accurately predict diseases for individuals outside that group. Such biases can arise from disparities in data collection, which can result in potential misdiagnoses or even missed diagnoses.

For example, if a wearable activity tracker shows varying accuracy levels between males and females, this could mean that the device is better calibrated for male users, meaning female users received less accurate health insights. This disparity could have arisen from an unrepresentative dataset during the device's calibration phase. Integrating wearable sensors and AI has significant potential for health data science. However, it is crucial to ensure that the datasets used are comprehensive, representative, and unbiased. Proper validation, continuous feedback, and inclusive design principles are essential to realising the full potential of this synergy.

## 8 How to address bias

Wearable sensors are devices such as fitness trackers, smartwatches, and smart clothing equipped with sensors that measure vital signs, physical activity, and other health-related metrics. Health data science is the intersection of data science and healthcare, focused on analysing health data to improve patient outcomes, optimise treatments, and reduce healthcare costs. For example, the Apple Watch uses green LED lights paired with light-sensitive photodiodes to detect the amount of blood flowing through the wrist at any given moment. This data is then processed to determine the heart rate.

Accelerometers are common components in wearable sensors used to measure movement and activity. Validating the accuracy of these sensors is crucial. Several publicly available datasets are used for accelerometer validation, including the MobiAct Dataset (31), which contains accelerometer data from smartphone sensors focused on human activity recognition, and the UCI HAR Datasets (40, 44–47, 57), collected from subjects performing activities of daily living (ADL) with a smartphone on their waist. Fitbit devices use accelerometers to track steps and activity levels, which feed into the device's algorithms to provide feedback and insights about the user's physical activity.

Biases can affect the data collection process, and the AI models generated, resulting in unfair or skewed outcomes. Therefore, it is crucial to identify and address any biases in both the data and algorithms. By doing so, wearable sensors and AI can offer unparalleled opportunities to predict, monitor, and improve people's health and well-being.

Several methods can be employed to ensure fairness in AI (64, 65). Firstly, it is essential to ensure that diverse participants are avoided, and that age, gender, ethnicity, and other biases are avoided during data collection. Secondly, the AI models should not propagate or amplify existing biases. Techniques like re-weighting training data, adversarial training, and fairness constraints can help to ensure algorithmic fairness. Lastly, regular audits of AI models can help detect, measure, and correct biases. Tools like Fairness Indicators can assist in evaluating the model's fairness metrics. For instance, in 2019, a study found gender and skin-type bias in commercial AI systems for analysing human emotions, indicating the need for improved fairness in these models.

The integration of wearable sensors and AI methods holds tremendous potential for healthcare. However, this potential can only be realised if the technology is accurate, reliable, and unbiased. By consistently collecting and validating data and conducting regular audits, we can harness the full power of this combination for the benefit of all.

To achieve this goal, manufacturers of wearable sensors must collaborate with AI researchers to ensure better integration and analysis. Establishing universal data collection and validation standards is crucial to ensure consistency. Continuous research and development are necessary to address biases in AI models effectively. We can only realise the full potential of wearable sensors and AI in healthcare.

## 9 Transparency and accountability

Wearable sensors are electronic devices embedded in watches, bands, and clothing. They can monitor physiological signals such as heart rate, temperature, and movement. With technological advancements, wearable sensors have become more accurate and efficient. Health data science is an interdisciplinary field that has emerged with the growth of wearable sensors. It focuses on extracting insights from complex and unstructured health data. Various ML and AI techniques are extensively used to predict, analyse, and understand health patterns derived from wearables. During the COVID-19 pandemic, wearable sensors have been instrumental in detecting early symptoms. Devices like the Oura Ring have been utilised to monitor temperature fluctuations and heart rate variability.

Accelerometers are integral components of many wearables and are used to track movement and activity. Validating the data produced by these sensors is essential for ensuring their reliability. Several datasets have been created specifically for this purpose, including the MobiAct Dataset, which recognises human activities such as walking, jogging, sitting, etc. The UCI HAR Dataset involves activities captured with a waist-mounted smartphone containing accelerometer and gyroscope sensors. The process of utilising wearable sensors and AI to gain insights into human health involves collecting raw data from wearables, cleaning it to remove noise and outliers, and then training AI and ML models on these datasets.

For instance, Stanford University launched the Wearable Health Lab in 2020, which uses algorithms to clean and pre-process data from wearables to ensure accurate health insights.

Transparency is a critical aspect of AI (18) in which clarity and openness of an AI system's operations are essential. Transparency helps to build trust (22, 66), and accountability and allows for refinement of AI systems (17–20, 67–69).

Google's DeepMind's AI for diabetic retinopathy detection is an excellent example of transparent operations. By making their algorithms transparent, doctors could trust and understand the diagnostic decisions made by the AI.

## 10 What is accountability?

Accountability in healthcare is regulated by national agencies, e.g., in the US, is HIPPA (70). However, since some of these regulations were created (e.g., the Health Insurance Portability and Accountability Act of 1996) healthcare services started integrating wearable sensors are devices designed to monitor and collect data about a user's physiological or environmental conditions. These sensors have become more popular, especially in the health and fitness sectors. Wearable sensors for health data science involve using technology to collect, process, and interpret health-related data. This data may include vital signs, activity levels, or environmental factors that could affect health.

Accelerometers are essential components in many wearables as they register physical activity, and ensuring data accuracy is crucial. Most wearable devices use accelerometers to capture motion-based data. These sensors detect changes in acceleration and can help deduce patterns of activity. Raw data from accelerometers often contains noise or irrelevant data points. Cleaning involves removing anomalies, while analysis transforms raw data into actionable insights, such as differentiating between walking, running, or sleeping. AI is also used for analysing large and complex datasets from wearable sensors. Specific models and techniques aid in data interpretation and decision-making. Neural Networks, especially recurrent networks (such as LSTM), are adept at processing time series data, which is abundant in wearable sensors. Decision trees are often used for classification tasks, such as discerning between different types of activities based on sensor data. Clustering Algorithms can be employed to identify patterns or groupings in large datasets, potentially uncovering health trends or anomalies.

Therefore, accountability in the context of AI refers to the responsibility to justify and explain AI systems' decisions (19). It is focused on ensuring transparency, fairness, and ethical use of AI in health wearables (65).

The following parties are accountable for ensuring ethical and responsible use of AI in health wearables:

1. Developers: AI model developers must ensure their models are designed fairly and ethically.
2. Manufacturers: Wearable manufacturers should ensure that their sensor data collection is accurate, and their AI processing is transparent.
3. Users: The onus is also on the users to use wearables responsibly and understand the implications of their data use and sharing.
4. Regulatory Bodies: Governments and organisations may set standards and regulations for AI's ethical and responsible use in wearable technology.

The confluence of wearable sensors and artificial intelligence has opened a promising frontier, particularly in health data science. Two examples of this being implemented in the real world include Fitbit and Apple's HealthKit.

Fitbit, a leading brand in wearable fitness trackers, processes vast amounts of data it collects through neural network architectures. This AI-driven analysis helps provide accurate health metrics and insights to its users. Similarly, Apple's HealthKit emphasises user privacy, ensuring that data collected by its wearable, Apple Watch, is stored securely. The onus is also on app developers integrating with HealthKit to ensure they follow Apple's stringent privacy guidelines.

## 11 Case study on transparency and accountability

Wearable sensors are devices people can wear to collect and transmit data related to their health, motion, or environment. These sensors can measure heart rate, body temperature, sleep patterns, and steps taken. With the rise of health-conscious consumers, there is a greater demand for devices that can provide real-time health monitoring. An accelerometer is a device used in wearable sensors to detect motion activities like walking, running, or climbing. Validation datasets help ensure that the data captured by the accelerometer is accurate and reliable. By comparing the results from wearable sensors with these datasets, manufacturers and health professionals can ascertain the devices' efficiency and reliability.

To collect data, raw data is acquired from wearable sensors, often stored in a local or cloud database. Data collection frequency and volume vary based on the device's purpose. Data cleaning is crucial to remove any anomalies or errors in the collected data. Data cleaning tools and techniques can help identify missing values, outliers, or incorrect data points that may skew analysis results. Once the data is cleaned, it is analysed using various algorithms and models. Artificial intelligence and machine learning play a pivotal role at this stage, helping to decipher patterns, anomalies, and insights from the data.

### 11.1 Artificial intelligence (AI) and machine learning (ML) models

AI and ML models are designed to recognise patterns, make decisions, and predict outcomes by learning from historical data. As a result, wearable devices can perform advanced tasks such as gait analysis, sleep pattern recognition, fall detection, and heart rate variability prediction.

Neural Networks, for instance, are algorithms that function similarly to the human brain. They can recognise patterns in data and identify subtle changes that may indicate a potential health problem. For example, a wearable device equipped with a neural network algorithm can detect changes in gait and alert the wearer of potential balance issues.

Decision Trees are another type of model used in wearable technology. They are particularly useful in classifying physical activities like running, walking, or cycling. By analysing data from sensors such as accelerometers and gyroscopes, the decision tree algorithm can accurately identify the type of activity the wearer is engaged in.

Support Vector Machines (SVMs) are also widely used in wearable technology to predict heart rate variability based on historical data. SVMs can be employed for regression and classification problems, making them a versatile model for wearable devices.

AI and ML models have improved wearable technology's ability to provide personalised and accurate data. This, in turn, has led to improved health and fitness outcomes for users. As technology advances, we can expect AI and ML models to play an increasingly important role in the development of wearable technology.

### 11.2 Case study on transparency and accountability: Fitbit's data usage

Fitbit, a leading wearable device company, was scrutinised for handling and using user data. With millions of users worldwide, Fitbit collects enormous amounts of personal health data daily. Concerns arose when it was revealed that user data might be sold to third parties, posing privacy risks. To address these concerns, Fitbit made efforts to be more transparent about its data usage policies. They ensured users that their data would not be sold for advertising purposes and implemented stricter data protection measures, giving users more control over their data. This case highlights the importance of transparency and accountability when handling sensitive personal data. It emphasises the need for wearable device companies to prioritise user trust and data protection. Integrating wearable sensors and artificial intelligence holds great promise for health data science. However, as technology advances, ethical data usage and privacy challenges arise. Ensuring transparency and accountability becomes paramount to maintaining user trust and ensuring the sustainable growth of this sector.

### 11.3 How to achieve transparency and accountability

Wearable sensors are electronic devices worn on the body as clothing or accessories. They are designed to measure specific physiological or environmental parameters such as heart rate, body temperature, or air quality. Health data science involves dealing with the large volumes of health data generated by wearable sensors. This data can provide invaluable insights into individual and population health when processed and analysed correctly. Accelerometers are among the most common wearable sensors. They measure acceleration forces and can infer movement patterns. Validating accelerometer datasets against known standards or ground truths is crucial to ensuring accuracy. Data collected from wearables often contain noise due to sensor misalignment, environmental factors, or system glitches. As a result, it becomes crucial to clean the data before processing. Once the data is cleaned, it can be processed using advanced analytics techniques, especially AI, to derive meaningful insights.

For instance, Fitbit and Apple Watch use accelerometers to track users' physical activity levels. Combined with AI, these devices can provide insights into a user's health and fitness journey. The integration of wearable sensors and AI has the potential to revolutionise healthcare, fitness, and many other sectors. However, as these technologies become more advanced, there is a need to ensure transparency, accountability, and ethical considerations. This is particularly important given the sensitive nature of the data collected by wearables. Explainable AI (XAI) is crucial to achieving transparency and accountability in AI (11). XAI aims to make the decision-making processes of AI systems understandable and interpretable by humans. This ensures that the logic behind AI decisions is clear and transparent.

Additionally, regulatory oversight and transparent governance must address ethical considerations and potential biases in AI models, particularly in healthcare. This ensures that AI models are trained on diverse datasets representative of the larger population and that the decisions are ethically sound.

Specific AI and ML models are suitable for wearable sensor data. CNN are perfect for accelerometer data because they suit time-series and spatial hierarchies. RNN and Long Short-Term Memory (LSTM) are designed to handle sequences, such as time-series data from wearables. Decision Trees and Random Forests are useful in making explicit decision paths, thus increasing transparency.

For example, using AI to predict cardiac events from wearable sensor data should be accurate and explainable. If a prediction is made, doctors, patients, and other stakeholders should understand the basis of that prediction, ensuring trust in the system. AI-driven solutions using wearable sensor data can be powerful and trustworthy if they implement explainable AI techniques and robust regulatory frameworks.

## 12 Privacy and data protection

Wearable and devices continuously monitor physiological and environmental parameters. They are portable and easy to use, making them popular among the public and the medical community. Health data science integrates techniques from statistics, computer science, and information theory to extract meaningful patterns from large datasets. These patterns can be used for early disease detection, fitness monitoring, or personalised medical treatments.

Privacy is essential in the context of wearable sensors. It refers to the right of individuals to keep their personal data and information secret and free from unauthorised access. Protecting privacy and confidentiality is critical as wearable devices capture sensitive information about an individual's health. It is essential to obtain user consent and make them aware of what their data will be used for. Organisations mishandling personal data can face severe penalties under laws like the General Data Protection Regulation (GDPR) (26, 27, 71–73).

Anonymising data doesn't always guarantee privacy (74), as AI techniques can sometimes reverse-engineer and identify individuals. Additionally, AI models, especially deep neural networks, are often seen as “black boxes.” This means that if these models are making healthcare decisions based on wearable sensor data, users must understand how these decisions are made.

Real-world examples of wearable sensor technology include the Apple Watch, which uses its built-in sensors to monitor heart rhythms and has an ECG app. While it has alerted users to potential health issues, it has also raised privacy concerns, given the intimate nature of the data collected. Fitbit is another example that collects data on steps, sleep, and other health metrics. The company's privacy policy indicates that it uses this data for targeted advertising, raising user concerns.

## 13 Data protection laws

AI and ML models are particularly effective in handling time-series data generally produced by wearable devices. Before applying AI, wearable data often needs noise reduction, normalisation, and imputation of missing values. Tools like pandas in Python provide capabilities for these tasks.

Specific datasets are commonly used for wearable sensor data analysis, e.g., MobiAct for activity recognition, UCI Human Activity Recognition Using Smartphones dataset for performing activities like walking and sitting while wearing a smartphone. These datasets provides both raw and pre-processed accelerometer data. Real-world examples of wearable sensor data analysis include the Apple Watch's fall detection feature, which uses accelerometer and gyroscope data to detect a hard fall and automatically alert emergency services if the wearer is unresponsive. Fitbit uses accelerometer data, along with other sensors, to detect sleep patterns, durations, and disturbances. This provides users with insights into their sleep health.

AI and ML have shown great potential in wearable sensor data analysis, and various models and techniques are available for handling different types of data. With the help of specific datasets and real-world examples, wearable devices can provide valuable insights into personal health and well-being.

Data Protection and Regulations are crucial in the field of wearable technology. The GDPR (26, 27) and the CCPA (75) are important regulations that emphasise users' rights over personal data. The GDPR (26, 27) is a regulation in the EU that requires wearable manufacturers and health data processors in the EU or serving EU residents to ensure strict adherence to users' rights over their personal data. The CCPA, on the other hand, is like GDPR but is specifically for California residents. It gives California consumers rights over their personal data, including knowing how it is used and the ability to opt out of data sales.

**Figure 3:**
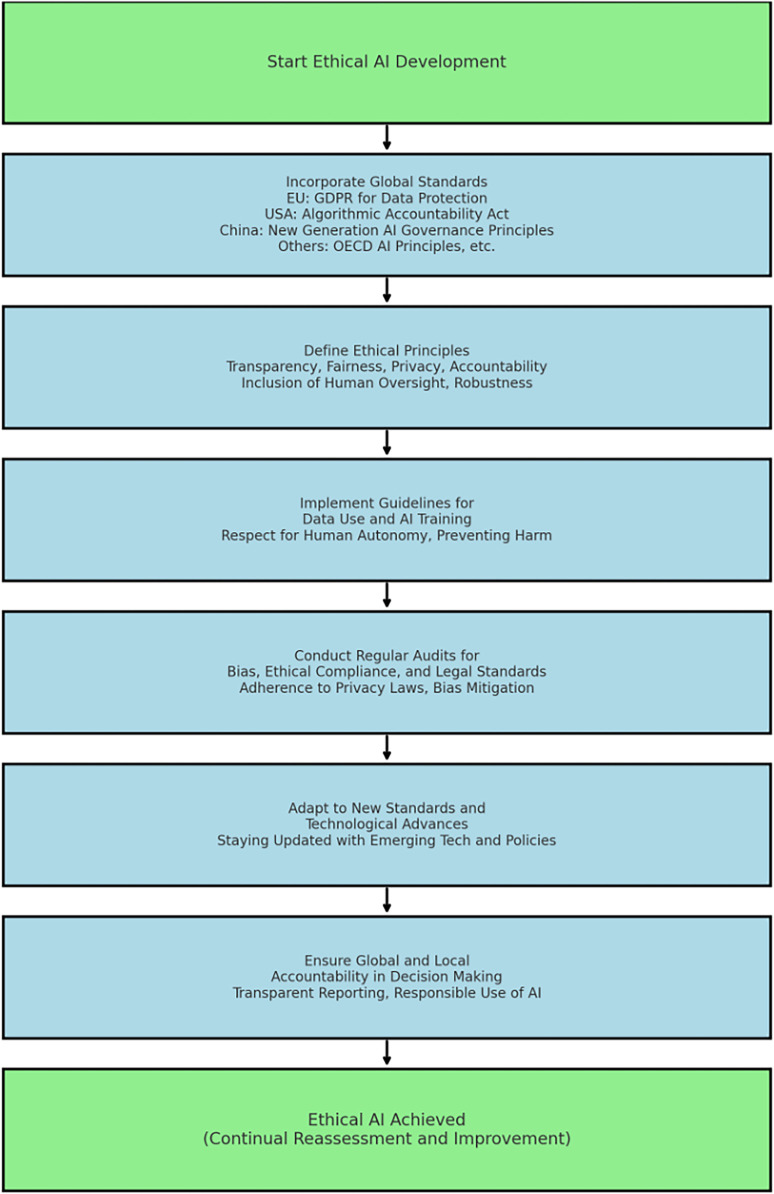
New model for compliant development of private, responsible and ethical AI.

In Figure 3, the fundamental principles of all standards and regulations discussed in this article are aligned with a universal and standardised approach for ethical AI development. This discussion is expanded into a new regulatory framework model with specific steps to ensure compliance with national and international regulatory frameworks, that guide responsible AI development.

Figure 3 presents a new model for the compliant development of private, responsible, and ethical AI in the context of wearable health technologies. This model outlines a stepwise progression through key development phases aimed at embedding regulatory alignment and ethical safeguards. The figure captures the procedural logic of regulatory compliance, to identify *transparency* as a discrete component of critical importance to each stage of ethical AI development. Transparency is a cornerstone of regulatory frameworks such as the GDPR (26, 71–74), the UK Data Protection Act (27), and the EU AI Act (28, 29), which all mandate clear documentation of data usage, explainability of algorithmic outputs, and auditability of automated decision systems. Without this, the claims of ethical compliance remain procedurally incomplete. Therefore, transparency must be recognised as both a transversal principle and a procedural requirement that legitimises each phase of the model.

Figure 3, presents a new model for ethical AI development. Given the sensitive nature of health data collected by wearables, adherence to GDPR, CCPA, and other regional regulations is paramount. The model and the process described in the Figure 3 are discussed in the context of protecting privacy.

Transparency undergirds the entire framework in Figure 3, and is indispensable to achieving the outcomes described in the model's final step: responsible and ethical AI. In particular, the steps toward compliance assume the existence of traceable data pathways, explainable algorithmic behaviour, and the ability to demonstrate adherence to user rights and accountability mechanisms. These assumptions mirror the obligations set out in Articles 13–15 of the GDPR (26, 27), which compel data controllers to provide individuals with meaningful information about automated decisions and their underlying logic. Moreover, the Ethics Guidelines for Trustworthy AI (42) explicitly identify transparency (including traceability, explainability, and communication) as a prerequisite for trust. Accordingly, any operationalisation of the model must embed transparency at each phase, not as a passive backdrop but as an active design principle. Without it, claims of ethical conformity lack verifiability and risk non-compliance with legal and normative standards governing AI systems in healthcare.

## 14 How to protect privacy

Popular datasets such as the Human Activity Recognition Using Smartphones Data Set (40, 44, 45), WISDM (Wireless Sensor Data Mining) (46, 54), and the Actitracker Dataset (52, 54), use a standard data privacy process. The data pipeline typically follows these stages: data collection, data cleaning, and analysis. Sensors continuously collect data about users, which is then cleaned using noise reduction techniques such as the Kalman or Low-pass filters. Outliers and irrelevant data points are removed, and ML models are used to categorise activities or detect anomalies. The question is, how is this process protecting privacy?

Anonymisation is a technique that modifies personal data so individuals cannot be easily identified. Some of the methods used in anonymisation are data masking, which replaces the original data with modified content (for example, “XXXX-1234” for a credit card number), generalisation, which reports age ranges instead of exact ages, and noise addition, which introduces random data to sensitive data points.

Secure data storage and transmission are also essential to ensure privacy and security. Data encryption is a technique used to transform data into an unreadable format unless decrypted with a key. AES (76) and RSA (77) are common encryption standards. Secure Socket Layer (SSL)/ Transport Layer Security (TLS) is another technique that ensures secure data transfer over networks. Also, blockchain technology is used for tamper-proof and decentralised data storage. Privacy in the data pipeline is also dependent on the methodology used to process and analyse the data.

## 15 Discussion on data-driven methodologies

Data-driven methodologies have become increasingly popular for extracting meaningful insights from large datasets. Wearable sensors have emerged as one of the most useful data sources, particularly in healthcare, fitness, and lifestyle areas. These sensors offer unique applications and data that can be used to gain valuable insights into various aspects of an individual's life.

**Figure 4:**
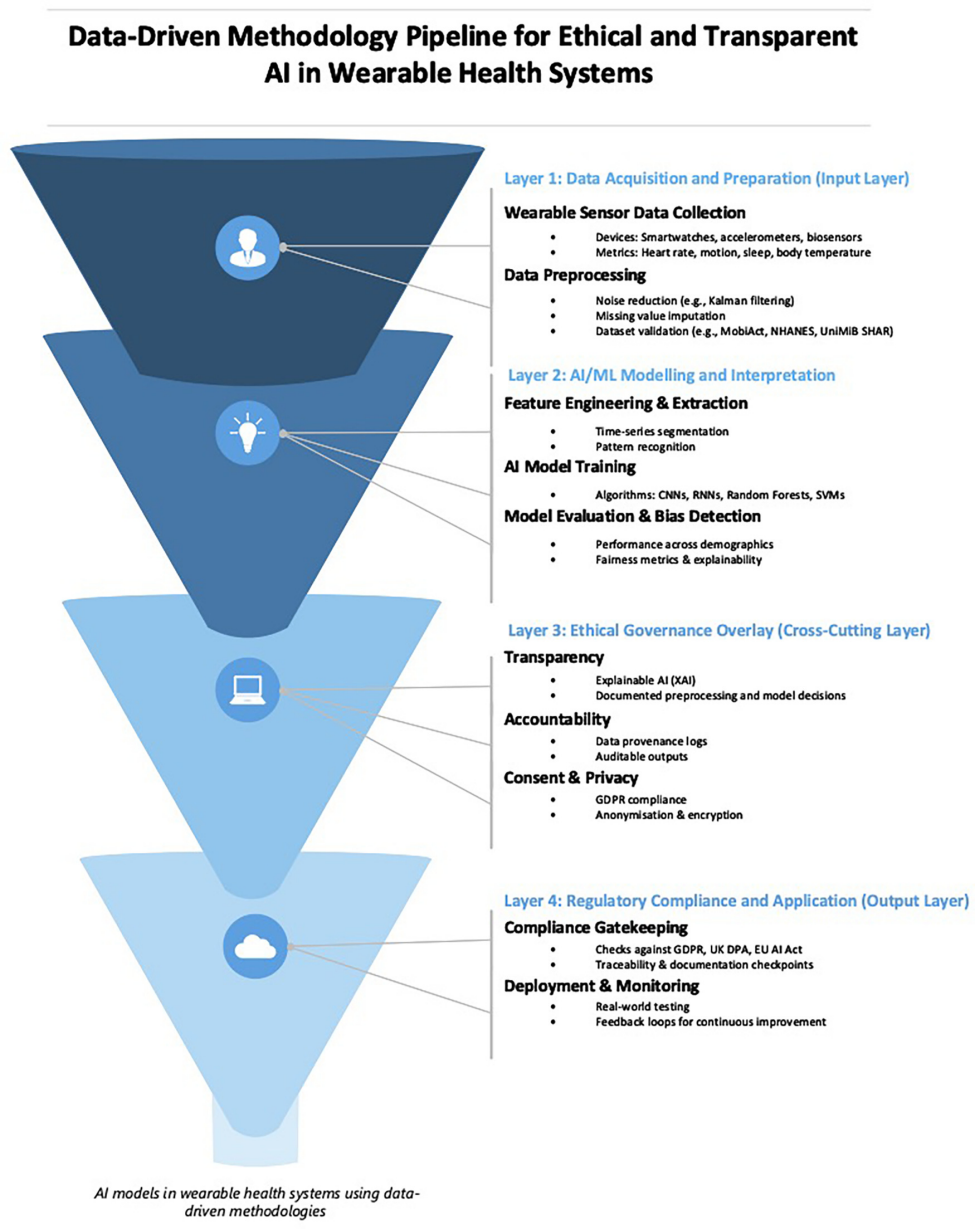
A structured framework for developing AI models in wearable health systems using data-driven methodologies.

Figure 4 presents a data-driven methodology pipeline for developing AI systems in wearable health technologies. The figure maps the end-to-end technical workflow (beginning with sensor data acquisition and ending with system deployment) while integrating ethical governance mechanisms across each phase. One key theme underpinning this pipeline is *transparency*, which, although embedded within the structure of the diagram, merits explicit discussion due to its foundational role in ensuring explainability, user trust, and regulatory compliance. As the review of recent literature on AI ethics (in earlier sections) has shown, transparency is a critical mechanism that enables traceability, enables informed consent, and reduces the opacity of algorithmic decision-making. Its inclusion as a horizontal, cross-cutting governance layer in the pipeline reflects its pervasive influence across data processing, modelling, and regulatory auditing activities.

In Figure 4, time-series analysis is one of the most popular data-driven methodologies (78, 79), for wearable sensors (80). This involves analysing continuous signals from wearables (81), like heart rate or step count over time. Time-series analysis can monitor a person's overall health, sleep patterns, or physical activity levels. Additionally, anomaly detection can detect abnormal patterns in heart rate, skin temperature, or other vital signs that may alert users or healthcare professionals to potential health issues. Pattern recognition and machine learning are other data-driven methodologies used with wearables. This approach recognises specific activities or movements, such as differentiating between walking, running, or cycling, using data from an accelerometer. Deep learning, especially CNN (37), processes and analyses multivariate time-series data, such as ECG signals (82), for detailed insights and predictions. Feature engineering and extraction (83) is a methodology (84, 85), used to transform raw data from sensors like accelerometers or gyroscopes into meaningful metrics like “steps taken” or “calories burned.” Predictive modelling is another methodology used to predict health events or outcomes, such as the risk of falling for elderly individuals, based on gait data.

Building upon these elements, in Figure 4, signal processing is another useful methodology that involves cleaning and enhancing sensor signals. This is used extensively in ECG, EMG, and EEG wearables to remove noise. Cluster analysis is a methodology used to group individuals based on their behaviour or health metrics, such as categorising sleep patterns. Finally, data fusion and multi-sensor integration combine data from multiple wearables or sensors to get a comprehensive view. For instance, using heart rate and GPS data to monitor an athlete's performance over varied terrains. Adding to this, applications of wearable sensors and data-driven methodologies constantly expand, leading to interdisciplinary collaborations between medicine, sports science, psychology, and engineering. This collaboration promises to bring more personalised and precise interventions in the future. Some useful wearable technology applications include biofeedback mechanisms, geospatial analysis, quantified self-movement, continuous monitoring and alerts, and behavioural analytics. Biofeedback mechanisms provide real-time feedback to users based on their physiological data and can guide meditation or relaxation exercises based on real-time heart rate variability. Geospatial analysis uses location data from wearables with GPS capabilities to study movement patterns, monitor outdoor activities, or even track disease spread in epidemiological studies. The quantified self-movement empowers individuals to monitor and analyse their personal data, from sleep cycles to mood fluctuations, for self-improvement and wellness.

Another element in Figure 4 is continuous monitoring alerts, which involve continuously tracking vital signs in at-risk patients. The wearable device can alert the user or a medical professional if certain predefined thresholds are breached. Behavioural analytics aim to understand user behaviour patterns and can be useful for personalised interventions or fitness recommendations. Finally, data privacy and security mechanisms ensure that data from wearables is securely stored and transmitted, given the data's personal and often sensitive nature.

While transparency is not isolated as a single terminal node in the Figure 4, it acts as a connective tissue binding each layer of the pipeline, from raw data handling to final deployment. The flow to the bottom step “Deployment & Monitoring”, assumes that upstream processes are explainable, documentable, and compliant with legal mandates such as Articles 13–15 of the GDPR (26, 27), which require data controllers to provide users with intelligible information about automated processing. Furthermore, the Ethics Guidelines for Trustworthy AI (42), and the UK's Data Protection Act (27) both frame transparency as a precondition for lawful, fair, and accountable AI use. In this diagram, transparency is visualised within the Ethical Governance Layer, highlighting its role in aligning technical development with institutional accountability and societal expectations. By explicitly situating transparency within this framework, the model ensures that data-driven innovation is not only methodologically robust but also normatively grounded.

## 16 Conclusion and future directions

This study has provided a detailed examination of the ethical, privacy, and regulatory challenges arising from the integration of AI and ML in wearable sensor technologies. The findings affirm that while these technologies offer transformative benefits (such as real-time health monitoring, predictive diagnostics, and personalised care) they also introduce a new class of risks related to algorithmic bias, data misuse, opaque decision-making, and the erosion of user autonomy.

Throughout the investigation, I identified systemic gaps in regulatory enforcement, inconsistencies in consent mechanisms, and the disproportionate impact of biased datasets on underrepresented populations. These issues are exacerbated by the black-box nature of many deep learning models, which hinders explainability and undermines user trust. Furthermore, the pervasive collection of sensitive biometric and behavioural data raises significant concerns around privacy, data sovereignty, and long-term data stewardship.

In response to these challenges, this study has proposed a data-driven methodological framework (outlined in Figure 4) that explicitly embeds transparency, accountability, and regulatory alignment across each phase of the AI development lifecycle. Unlike previous conceptual models, this framework is built with transparency as a technical and procedural necessity. It integrates transparency into system design, from raw data collection to real-world deployment, thereby operationalising legal obligations set forth by instruments such as the GDPR, the UK Data Protection Act, and the EU AI Act.

The model advances a layered approach that connects data provenance, explainability, and compliance auditing through a horizontal ethical governance layer. This structural integration ensures that AI models trained on wearable sensor data are not only performant but also defensible in contexts of public scrutiny, legal accountability, and institutional ethics review. It reflects a shift toward normative design practices that embed compliance, fairness, and inclusivity as core development goals, rather than *post hoc* considerations.

Looking ahead, future research and regulatory development must focus on four key challenges: (1) enhancing privacy safeguards across distributed AI systems; (2) improving informed consent through intelligible, user-centric interfaces; (3) detecting and mitigating algorithmic bias in real-time deployments; and (4) enforcing transparency requirements in complex AI architectures, especially in deep learning contexts. Addressing these challenges is essential to ensure legal compliance and to cultivate societal trust and unlock the full potential of AI-enabled wearables in healthcare.

## References

[1] Helbing D Frey BS Gigerenzer G Hafen E Hagner M Hofstetter Y Will democracy survive big data and artificial intelligence? In: Helbing D, editor. Towards Digital Enlightenment: Essays on the Dark and Light Sides of the Digital Revolution. Zurich: Springer International Publishing (2018). p. 73–98. 10.1007/978-3-319-90869-4_7

[2] Bécue A Praça I Gama J. Artificial intelligence, cyber-threats and industry 4.0: challenges and opportunities. Artif Intell Rev. (2021) 54(5):3849–86. 10.1007/S10462-020-09942-2

[3] Hernandez N Lundström J Favela J McChesney I Arnrich B. Literature review on transfer learning for human activity recognition using mobile and wearable devices with environmental technology. SN Computer Science. (2020) 1(2):1–16. 10.1007/s42979-020-0070-4

[4] Jiang F Jiang Y Zhi H Dong Y Li H Ma S Artificial intelligence in healthcare: past, present and future. Stroke Vasc Neurol. (2017) 2(4):230–43. 10.1136/SVN-2017-000101 29507784 PMC5829945

[5] Bartoletti I. AI in healthcare: ethical and privacy challenges. In: Riaño D Wilk S ten Teije A, editors. Lecture Notes in Computer Science (Including Subseries Lecture Notes in Artificial Intelligence and Lecture Notes in Bioinformatics). Vol. 11526. Cham: Springer (2019). p. 7–10. 10.1007/978-3-030-21642-9_2

[6] Cossy-Gantner A Germann S Schwalbe NR Wahl B. Artificial intelligence (AI) and global health: how can AI contribute to health in resource-poor settings? BMJ Glob Health. (2018) 3(4):1–7. 10.1136/bmjgh-2018-000798 PMC6135465 30233828

[7] Shaheen MY. AI In healthcare: medical and socio-economic benefits and challenges. ScienceOpen Preprints. (2021). 10.14293/S2199-1006.1.SOR-.PPRQNI1.V1

[8] Shaheen MY. Applications of artificial intelligence (AI) in healthcare: a review. ScienceOpen Preprints. (2021). 10.14293/S2199-1006.1.SOR-.PPVRY8K.V1

[9] Liberati N. The borg–eye and the we–I. The production of a collective living body through wearable computers. AI & Soc. (2020) 35(1):39–49. 10.1007/s00146-018-0840-x

[10] Chen D Wu Y. Research on the use of communication big data and AI artificial intelligence technology to construct telecom fraud prevention behavior portrait. Intell Decis Technol. (2024) 18(3):1–17. 10.3233/IDT-240386

[11] Pawar U O’Shea D Rea S O’Reilly R. Explainable AI in healthcare. 2020 International Conference on Cyber Situational Awareness, Data Analytics and Assessment, Cyber SA; Dublin, Ireland (2020). 10.1109/CYBERSA49311.2020.9139655

[12] Ravi N Chaturvedi P Huerta EA Liu Z Chard R Scourtas A FAIR Principles for AI models with a practical application for accelerated high energy diffraction microscopy. Sci Data. (2022) 9(1):1–9. 10.1038/s41597-022-01712-9 36357431 PMC9649764

[13] Panch T Mattie H Celi LA. The “inconvenient truth” about AI in healthcare. NPJ Digital Medicine. (2019) 2(1):1–3. 10.1038/s41746-019-0155-4 31453372 PMC6697674

[14] Chen RJ Wang JJ Williamson DFK Chen TY Lipkova J Lu MY Algorithmic fairness in artificial intelligence for medicine and healthcare. Nat Biomed Eng. (2023) 7(6):719–42. 10.1038/s41551-023-01056-8 37380750 PMC10632090

[15] Huyen NTM Bao TQ. Advancements in AI-driven cybersecurity and comprehensive threat detection and response. J Intell Connectivity Emerg Technol. (2024) 9(1):1–12. Available at: https://questsquare.org/index.php/JOUNALICET/article/view/37

[16] Sebastian G. Do ChatGPT and other AI chatbots pose a cybersecurity risk?: an exploratory study. Int J Security Privacy Perv Comput. (2023) 15:1–11. 10.4018/IJSPPC.320225

[17] Springett S. Vulnerability and Exploitability Transparency - VDR & VEX | OWASP Foundation. OWASP (2023). Available at: https://owasp.org/blog/2023/02/07/vdr-vex-comparison (Accessed April 25, 2023).

[18] de Fine Licht K de Fine Licht J. Artificial intelligence, transparency, and public decision-making. AI Soc. (2020) 35:1–10. 10.1007/s00146-020-00960-w

[19] Raji ID Smart A White RN Mitchell M Gebru T Hutchinson B Closing the AI accountability gap: defining an end-to-end framework for internal algorithmic auditing. FAT* 2020 - Proceedings of the 2020 Conference on Fairness, Accountability, and Transparency (2020). p. 33–44. 10.1145/3351095.3372873

[20] Terzi S Stamelos I. Architectural solutions for improving transparency, data quality, and security in eHealth systems by designing and adding blockchain modules, while maintaining interoperability: the eHDSI network case. Health Technol (Berl). (2024) 14(3):451–62. 10.1007/S12553-024-00833-Y/FIGURES/6

[21] Bulling A Roggen D Tröster G. Wearable EOG goggles: Seamless sensing and context-awareness in everyday environments (n.d.).

[22] Turilli M Floridi L. The ethics of information transparency. Ethics Inf Technol. (2009) 11(2):105–12. 10.1007/s10676-009-9187-9

[23] NIST. Department of Commerce Announces New Guidance, Tools 270 Days Following President Biden’s Executive Order on AI. NIST (2024). Available at: https://www.nist.gov/news-events/news/2024/07/department-commerce-announces-new-guidance-tools-270-days-following (Accessed August 29 2024).

[24] NIST. AI Standards. NIST (2024). Available at: https://www.nist.gov/artificial-intelligence/ai-standards (Accessed August 29 2024).

[25] NIST. AI Risk Management Framework. NIST (2024). Available at: https://www.nist.gov/itl/ai-risk-management-framework (Accessed August 29 2024).

[26] GDPR. What is GDPR, the EU’s new Data Protection law? GDPR.eu (2018). Available at: https://gdpr.eu/what-is-gdpr/ (Accessed July 7, 2023).

[27] ICO. Information Commissioner’s Office (ICO): The UK GDPR. UK GDPR Guidance and Resources (2018). Available at: https://ico.org.uk/for-organisations/data-protection-and-the-eu/data-protection-and-the-eu-in-detail/the-uk-gdpr/ (Accessed July 8 2023)

[28] Bommasani R Klyman K Zhang D Liang P. Do Foundation Model Providers Comply with the Draft EU AI Act? Stanford Center for Research on Foundation Models (2023). Available at: https://crfm.stanford.edu/2023/06/15/eu-ai-act.html (Accessed May 29, 2025).

[29] European Parliament. AI Act: A Step Closer to the First Rules on Artificial Intelligence. News | European Parliament (2023). Available at: https://www.europarl.europa.eu/news/en/press-room/20230505IPR84904/ai-act-a-step-closer-to-the-first-rules-on-artificial-intelligence (Accessed July 7, 2023).

[30] Vavoulas G Pediaditis M Spanakis EG Tsiknakis M. The MobiFall dataset: an initial evaluation of fall detection algorithms using smartphones. 13th IEEE International Conference on BioInformatics and BioEngineering, IEEE BIBE 2013 (2013). 10.1109/BIBE.2013.6701629

[31] Vavoulas G Chatzaki C Malliotakis T Pediaditis M Tsiknakis M. The MobiAct dataset: recognition of activities of daily living using smartphones. ICT4AWE 2016 - 2nd International Conference on Information and Communication Technologies for Ageing Well and e-Health, Proceedings (2016). p. 143–51. 10.5220/0005792401430151

[32] Blanco-Filgueira B Garcia-Lesta D Fernandez-Sanjurjo M Brea VM Lopez P. Deep learning-based multiple object visual tracking on embedded system for IoT and mobile edge computing applications. IEEE Internet of Things Journal. (2019) 6(3):5423–31. 10.1109/JIOT.2019.2902141

[33] Alex A Krizhevsky K Ilya I Sutskever S Hinton GE. ImageNet Classification Classification with Deep Convolutional Convolutional Neural Networks. Pdfs.Semanticscholar.Org (n.d.). Available at: https://pdfs.semanticscholar.org/3cd5/a85dc9da55dc0b7aa7787ba49925f79b32e6.pdf (Accessed October 20, 2023).

[34] Platt J. C. (n.d.). Probabilistic Outputs for Support Vector Machines and Comparisons to Regularized Likelihood Methods. Available at: https://www.researchgate.net/publication/2594015 (Accessed September 3, 2023).

[35] DeepDance model. DeepDance: Music-to-Dance Motion Choreography with Adversarial Learning. GitHub (2021). Available at: https://github.com/computer-animation-perception-group/DeepDance (Accessed March 16 2025).

[36] Jobin A Ienca M Vayena E. The global landscape of AI ethics guidelines. Nature Machine Intelligence. (2019) 1(9):389–99. 10.1038/s42256-019-0088-2

[37] Krizhevsky A Sutskever I Hinton GE. Imagenet classification with deep convolutional neural networks. Commun ACM. (2017) 60(6):84–90. 10.1145/3065386

[38] Jang M Seo S Kang P. Recurrent neural network-based semantic variational autoencoder for sequence-to-sequence learning. Inf Sci (Ny). (2019) 490:59–73. 10.1016/J.INS.2019.03.066

[39] Haque MR Islam MM Iqbal H Reza MS Hasan MK. Performance evaluation of random forests and artificial neural networks for the classification of liver disorder. International Conference on Computer, Communication, Chemical, Material and Electronic Engineering, IC4ME2 2018 (2018). 10.1109/IC4ME2.2018.8465658

[40] Micucci D Mobilio M Napoletano P. Unimib SHAR: a dataset for human activity recognition using acceleration data from smartphones. Applied Sciences (Switzerland. (2017) 7(10):1–19. 10.3390/APP7101101

[41] UniMiB SHAR. (2023). NHANES Questionnaires, Datasets, and Related Documentation. Available at: https://wwwn.cdc.gov/nchs/nhanes/ (Accessed March 16, 2025).

[42] European Commission. Ethics Guidelines for Trustworthy AI. Shaping Europe’s digital future (2018). Available at: https://digital-strategy.ec.europa.eu/en/library/ethics-guidelines-trustworthy-ai (Accessed July 7, 2023).

[43] Röösli E Rice B Hernandez-Boussard T. Bias at warp speed: how AI may contribute to the disparities gap in the time of COVID-19. J Am Med Inform Assoc. (2021) 28(1):190–2. 10.1093/jamia/ocaa210 32805004 PMC7454645

[44] UCI. Human Activity Recognition Using Smartphones, Machine Learning Repository, Human Activity Recognition Using Smartphones Dataset. (2012). Available at: https://archive.ics.uci.edu/dataset/240/human+activity+recognition+using+smartphones (Accessed 16 March 2025).

[45] Anguita D Ghio A Oneto L Parra X Reyes-Ortiz JL. A public domain dataset for human activity recognition using smartphones. The European Symposium on Artificial Neural Networks (2013).

[46] WISDM. Human Activity Recognition on the Wireless Sensor Data Mining (WISDM) Dataset Using Convolutional Neural Network and Convolutional Autoencoder. GitHub (2022). Available at: https://github.com/topics/wireless-sensor-data-mining (Accessed March 16, 2025).

[47] HHAR. Heterogeneity Dataset for Human Activity Recognition (HHAR). GitHub (2017). Available at: https://github.com/Limmen/Distributed_ML (Accessed March 16, 2025).

[48] Stisen A Blunck H Bhattacharya S Prentow TS Kjærgaard MB Dey A Smart devices are different: assessing and mitigating mobile sensing heterogeneities for activity recognition. SenSys 2015 - Proceedings of the 13th ACM Conference on Embedded Networked Sensor Systems (2015). p. 127–40. 10.1145/2809695.2809718

[49] PAMAP2. PAMAP2 Physical Activity Monitoring - UCI Machine Learning Repository. (2012). Available at: https://archive.ics.uci.edu/dataset/231/pamap2+physical+activity+monitoring (Accessed March 16 2025).

[50] Reiss A Stricker D. Introducing a new benchmarked dataset for activity monitoring. International Semantic Web Conference (2012). p. 108–9. 10.1109/ISWC.2012.13

[51] UCI. Daphnet Freezing of Gait - UCI Machine Learning Repository (2013). Available at: https://archive.ics.uci.edu/dataset/245/daphnet+freezing+of+gait (Accessed March 16, 2025).

[52] Actitracker. Human Activity Recognision Using Convolution Neural Network. GitHub (2017). Available at: https://github.com/gomahajan/har-actitracker (Accessed March 16, 2025).

[53] Kwapisz JR Weiss GM Moore SA. Activity Recognition using Cell Phone Accelerometers (2010).

[54] Actitracker Dataset. WISDM Lab: Dataset. WISDM Lab (2013). Available at: https://www.cis.fordham.edu/wisdm/dataset.php (Accessed March 16, 2025).

[55] UCI. Daily and Sports Activities - UCI Machine Learning Repository. (2013). Available at: https://archive.ics.uci.edu/dataset/256/daily+and+sports+activities (Accessed March 16, 2025).

[56] Altun K Barshan B Tunçel O. Comparative study on classifying human activities with miniature inertial and magnetic sensors. Pattern Recogn. (2010) 43(10):3605–20. 10.1016/J.PATCOG.2010.04.019

[57] Davis K Evans O. Smartphone Dataset for Human Activity Recognition (HAR) in Ambient Assisted Living (AAL). UCI Machine Learning Repository (2016). 10.24432/C5P597

[58] Roggen D Calatroni A Nguyen-Dinh LV Van C Ricardo SH. Opportunity Activity Recognition - UCI Machine Learning Repository. Irvine, CA: GitHub, University of California (2012). 10.24432/C5M027

[59] Roggen D Calatroni A Rossi M Holleczek T Förster K Tröster G Collecting complex activity datasets in highly rich networked sensor environments. International Conference on Networked Sensing Systems (2010). p. 233–40 10.1109/INSS.2010.5573462

[60] CASAS Datasets. Center for Advanced Studies in Adaptive Systems. Washington: Washington State University (2024). Available at: https://casas.wsu.edu/datasets/ (Accessed March 16, 2025).

[61] Li W. MSR Action 3D. Microsoft Research (2014). Available at: https://wangjiangb.github.io/my_data.html (Accessed March 16, 2025).

[62] Oresti Banos Mate Toth Oliver Amft. REALDISP (REAListic Sensor DISPlacement) Dataset: Activity Recognition Dataset - UCI Machine Learning Repository.Irvine, CA: University of California (2014). 10.24432/C5GP6D

[63] Castres I Tourny C Lemaître F Coquart J. Impact of a walking program of 10,000 steps per day and dietary counseling on health-related quality of life, energy expenditure and anthropometric parameters in obese subjects. J Endocrinol Investig. (2017) 40(2):135–41. 10.1007/s40618-016-0530-9 27600387

[64] Shu Y Zhang J Yu H. Fairness in design: a tool for guidance in ethical artificial intelligence design. In: Lecture Notes in Computer Science (including subseries Lecture Notes in Artificial Intelligence and Lecture Notes in Bioinformatics). Cham: Springer (2021). p. 500–10. 10.1007/978-3-030-77626-8_34

[65] Bender EM Gebru T McMillan-Major A Shmitchell S. On the dangers of stochastic parrots: can language models be too big? Proceedings of the 2021 ACM Conference on Fairness, Accountability, and Transparency (2021). p. 610–23 10.1145/3442188.3445922

[66] Poitras G Meredith L. Ethical transparency and economic medicalization. J Bus Ethics. (2009) 86(3):313–25. 10.1007/s10551-008-9849-2

[67] NTIA. SBOM at a Glance. NTIA Multistakeholder Process on Software Component Transparency. Ntia.Gov/Sbom (2021). Available at: https://tiny.cc/SPDX (Accessed January 3, 2023).

[68] NTIA. Survey of Existing SBOM Formats and Standards-Version 2021 Survey of Existing SBOM Formats and Standards Credit: Photo by Patrick Tomasso on Unsplash NTIA Multistakeholder Process on Software Component Transparency Standards and Formats Working Group. (2021). Available at: https://www.ntia.gov/files/ntia/publications/sbom_formats_survey-version-2021.pdf (Accessed December 25, 2022).

[69] Royce E. R. H.R.5793 - 113 Congress (2013-2014): Cyber Supply Chain Management and Transparency Act of 2014. Congress.Gov. (2014). Available at: http://www.congress.gov/ (Accessed January 3, 2023).

[70] HIPAA. Health Insurance Portability and Accountability Act of 1996 (HIPAA). CDC (1996). Available at: https://www.cdc.gov/phlp/publications/topic/hipaa.html (Accessed July 7, 2023).

[71] Li H Yu L He W. The impact of GDPR on global technology development. J Glob Inf Technol Manag. (2019) 22(1):1–6. 10.1080/1097198X.2019.1569186

[72] Hintze M El Emam K. Comparing the benefits of pseudonymisation and anonymisation under the GDPR. J Data Prot Priv. (2018) 2(2):145–58. 10.69554/QSST9019

[73] Peloquin D DiMaio M Bierer B Barnes M. Disruptive and avoidable: gDPR challenges to secondary research uses of data. Eur J Hum Genet. (2020) 28(6):697–705. 10.1038/s41431-020-0596-x 32123329 PMC7411058

[74] Shehu A-S. On the compliance of self-sovereign identity with GDPR principles: a critical review. J Latex Class Files. (2024) 14(8). Available at: https://arxiv.org/abs/2409.03624v1

[75] CCPA. California Consumer Privacy Act (CCPA). State of California - Department of Justice - Office of the Attorney General (2018). Available at: https://oag.ca.gov/privacy/ccpa (Accessed September 20, 2023).

[76] Daemen J Rijmen V. Note on naming Rijndael as the AES. (2003). Available at: http://csrc.nist.gov/CryptoToolkit/aes/rijndael/Rijndael.pdf (Accessed March 19, 2023).

[77] Fujita T Kogiso K Sawada K Shin S. Security enhancements of networked control systems using RSA public-key cryptosystem. 2015 10th Asian Control Conference: Emerging Control Techniques for a Sustainable World (2015). 10.1109/ASCC.2015.7244402

[78] LeCun Y Bengio Y. Convolutional networks for images, speech, and time series. The Handbook of Brain Theory and Neural Networks. (1995) 3361(10):1995.

[79] Niu WJ Feng ZK Feng BF Xu YS Min YW. Parallel computing and swarm intelligence based artificial intelligence model for multi-step-ahead hydrological time series prediction. Sustain Cities Soc. (2021) 66:102686. 10.1016/j.scs.2020.102686

[80] Krentz T Dubey A Karsai G. Short paper: towards an edge-located time-series database. Proceedings - 2019 IEEE 22nd International Symposium on Real-Time Distributed Computing, ISORC 2019 (2019). p. 151–4. 10.1109/ISORC.2019.00037

[81] Dhivyaprabha TT Subashini P Krishnaveni M Santhi N Sivanpillai R Jayashree G. A novel synergistic fibroblast optimization based kalman estimation model for forecasting time-series data. Evol Syst. (2019) 10(2):205–20. 10.1007/S12530-018-9217-0

[82] Porambage P Kumar T Liyanage M Partala J Lovén L Ylianttila M Sec-EdgeAI: AI for Edge Security Vs Security for Edge AI BrainICU-Measuring brain function during intensive care View project ECG-based emotion recognition View project Sec-EdgeAI: AI for Edge Security Vs Security for Edge AI. (2019). Available at: https://www.researchgate.net/publication/330838792 (Accessed October 28, 2019).

[83] Khurana U Nargesian F Samulowitz H Khalil EB Turaga D. Learning feature engineering for classification. (2017). 10.24963/ijcai.2017/352

[84] Khurana U Samulowitz H Turaga D. Feature engineering for predictive modeling using reinforcement learning. In Proceedings of the AAAI Conference on Artificial Intelligence. (2018) 32:1. 10.1609/aaai.v32i1.11678

[85] Khurana U Turaga D Samulowitz H Parthasrathy S. Cognito: automated feature engineering for supervised learning. IEEE International Conference on Data Mining Workshops, ICDMW (2016). p. 1304–7. 10.1109/ICDMW.2016.0190

